# Synergistic delivery of resveratrol and ultrasmall copper-based nanoparticles by aptamer-functionalized ultrasound nanobubbles for the treatment of nonalcoholic fatty liver disease

**DOI:** 10.3389/fphys.2022.950141

**Published:** 2022-09-09

**Authors:** Xinmin Guo, Zhihui Huang, Jialin Chen, Kun He, Jianru Lin, Hui Zhang, Yanying Zeng

**Affiliations:** ^1^ Department of Ultrasound, Guangzhou Red Cross Hospital of Jinan University, Guangzhou, China; ^2^ Department of Nuclear Medicine, Guangzhou Red Cross Hospital of Jinan University, Guangzhou, China

**Keywords:** ultrasonic, nanobubble, resveratrol, ultrasmall, copper-based, nanoparticles, nonalcoholic fatty liver

## Abstract

Nonalcoholic fatty liver disease (NAFLD) is related to the production of reactive oxygen species (ROS) and oxidative stress, so antioxidant treatment can prevent its further development. Ultrasmall copper-based nanoparticles (CuNPs) have shown multiple enzyme-like activities for scavenging oxygen species, providing a new strategy for the treatment of inflammatory diseases. Resveratrol (Res), a natural polyphenol compound, has attracted much attention due to its ability to inhibit oxidative stress. We therefore aimed to first combine these two agents for the treatment of NAFLD. However, due to the poor water solubility and stability of Res, which is easily metabolized in the intestine, the development of a stable and effective carrier became the key to achieving a synergistic effect. Liver-targeted nanocarriers loaded with bioactive compounds may provide a more effective approach for the treatment of NAFLD. Therefore, we developed a novel ultrasonic nanobubble carrying nucleic acid aptamers with liver targeting properties, which has the advantages of a small molecular weight, no immunogenicity, a low cost of synthesis, and high stability through chemical modification. Res and the ultrasmall CuNPs were specifically delivered to liver tissue to maximize therapeutic efficiency. This study found that the combination of these two components can effectively treat inflammation in NAFLD and suggested that liver-targeted NAFLD-specific aptamer-mediated targeted ultrasound nanobubbles that can simultaneously deliver Res and CuNPs may be a safe and effective new platform for NAFLD and other liver diseases.

## 1 Introduction

Nonalcoholic fatty liver disease (NAFLD) is now considered one of the most common chronic liver diseases, affecting approximately 25%–30% of people worldwide (Chachay et al., 2014; Berman et al., 2017). Its manifestations range from simple steatosis to liver injury, followed by liver fibrosis liver cirrhosis and liver cancer development, and a series of diseases, so early intervention has clinical significance (Kutlu et al., 2018). Recent studies have shown that NAFLD is associated with excessive production of reactive oxygen species (ROS) and oxidative stress, and hepatocytes are eventually damaged by oxidative stress and lipid peroxidation (Schwimmer, 2007; Lomonaco et al., 2013; Braud et al., 2017; Kasper et al., 2021; Ding et al., 2022).

At present, there is still a lack of effective therapeutic drugs for NAFLD in the clinic. From the perspective of pathogenic factors, the use of antioxidants can regulate adipogenesis, lipid oxidation and peroxidation, and inflammation. By improving the oxidative environment, liver damage and fibrosis may be improved, ultimately delaying, preventing, and reversing the progression of nonalcoholic steatohepatitis (NASH) and improving clinical outcomes. Many studies have shown that resveratrol (Res) (Abba et al., 2015; Rauf et al., 2017; Öztürk et al., 2017) has pharmacological effects on the nervous system, liver, and cardiovascular system and can reduce liver pathological damage by acting as an antioxidative stress factor. Recent research showed that ultrasmall copper-based nanoparticles have a variety of enzymatic activities and the ability to scavenge a variety of ROS and treat ROS-related diseases. At the same time, their ultrasmall size ensures that their strong ROS scavenging ability will reach any organ and might be rapidly subjected to renal clearance, avoiding damage to normal tissues. The synergy between these two drugs is expected to exert a strong antioxidative stress effect, but Res has poor water solubility and is not stable and easily metabolized by in intestinal tract, which limits its further clinical applications. Therefore, how to use an effective carrier to improve its stability and bioavailability is a current research hotspot (Francioso et al., 2014; Zupančič et al., 2015). In recent years, nanobased drug delivery systems have become good drug and gene carrier systems because of their stability and novel physical properties; they can quickly reach the target tissue through the vascular endothelial system. Some scholars reported that the use of poly(lactic-co-glycolic acid) (PLGA) nanoparticles as a carrier system for Res successfully achieved curative effects for nonalcoholic fatty liver (Wan et al., 2018).

Ultrasonic nanobubble-based drug delivery systems have attracted the attention of researchers because of their small size, excellent stability, and novel physical and surface properties, which allow them to quickly pass through the vascular endothelium and enter the target tissue for aggregation (Suzuki et al., 2016; Endo-Takahashi and Negishi, 2020; Sun et al., 2020). Although this type of nanobubble has the ability to achieve ultrasonic targeted release, the targeting specificity needs to be improved. At present, targeting is mainly achieved through the modification of specific targeting groups, such as antibodies, folic acid, and aptamers (Zhu et al., 2018; Shen et al., 2019; Yu et al., 2020). Nucleic acid aptamers have attracted much attention because of their unique performance advantages, such specific affinity equal to or even higher than that of antibodies, high stability, and high cost-effectiveness for mass production. Some scholars have constructed aptamer-modified nanobubbles for ultrasound imaging of various tumor cells (Zhu et al., 2018). To date, there are no reports on the synthesis of aptamer-modified nanobubbles loaded with drugs and nanoparticles for the treatment of NAFLD.

In this context, this study aimed to synthesize a nanobubble using thin-film hydration and mechanical oscillation, and the nanobubbles were modified with NAFLD-specific aptamers and loaded with Res and ultrasmall copper-based nanoparticles for ultrasonic targeted therapy for NAFLD. This study is expected to provide new ideas and a basis for the synergistic treatment of NAFLD with aptamer-functionalized nanobubbles for the delivery of drugs and nanoparticles.

## 2 Materials and methods

### 2.1 Main materials and instruments

The HepG2 human hepatoma cell line was purchased from the American Type Culture Collection (ATCC), and anhydrous copper chloride, L-ascorbic acid and sodium hydroxide were purchased from Sinopharm Group. Perfluoropropane was purchased from Sichuan 273 Degrees Environmental Protection Technology Co., Ltd.; DSPE-mPEG2K, DSPE-mPEGK-Mal and DOTAP were purchased from Qiyue Biological Co., Ltd.; Res was purchased from McLean; and lecithin was purchased from Aladdin. PBS was obtained from Gibco. The sulfhydryl-modified NAFLD01 aptamer sequence was purchased from Shanghai Shenggong Biological Co., Ltd. Other reagents not specifically mentioned were analytically pure. The magnetic stirrer (Gongyi Yuhua Instrument Co., Ltd.), centrifuge (Pingfan Technology Co., Ltd.), rotary evaporator (Changchun Technology Co., Ltd.), cell breaker (Ningbo Xinzhi Bio), multifunctional microplate reader (Tecan), laser nanoparticle sizer (Brookhaven), and ultraviolet absorber (Shanghai Jinghua Technology Co., Ltd.), X-ray diffraction (XRD) diffractometer, Rigaku D/max 2550) used in the study were purchased from Rigaku Company of Japan. A transmission electron microscope (FEI Company of America), X-ray photoelectron spectrometer (Thermo Fisher, ESCALAB 250Xi), fluorescence spectrometer (Hitachi, Japan), and inductively coupled plasma atomic emission spectrometer (ICP–AES, 7000DV, PerkinElmer) were also used.

### 2.2 Experimental methods

#### 2.2.1 Establishment of a cell model of nonalcoholic fatty liver induced by high-oil fatty acids

The human hepatocellular carcinoma cell line HepG2 was obtained from the ATCC. Cells were cultured in Dulbecco’s modified Eagle’s medium containing 10% fetal bovine serum without penicillin–streptomycin. HepG2 cells were cultured with free fatty acid (FFA) DMEM for approximately 48 h. FFA components were composed of palmitic acid and oleic acid (Sigma-Aldrich) at a ratio of 1:2 and diluted with FBS-free DMEM to a final concentration of 4 mM. The cells were stained with oil red O to confirm fat deposition (Pu et al., 2021).

#### 2.2.2 Evaluation of the targeted binding ability of aptamers in the context of nonalcoholic fatty liver disease

##### 2.2.2.1 Laser confocal microscopy experiment

The groups were as follows. There were five groups in total: ① NAFLD cells + NAFLD01 aptamer sequence; ② NAFLD cells + nonsense aptamer sequence; ③ HepG2 cells + NAFLD01 aptamer sequence; ④ human normal hepatocytes + NAFLD01 aptamer sequence; and ⑤ human embryonic fibroblasts + NAFLD01 aptamer sequence. The methods were as follows. HepG2 cells were cultured in accordance with the above methods. First, oil red O staining was performed to confirm fat deposition. Then, NAFLD01 aptamer and nonsense aptamer sequences labeled with 250 nM FITC were incubated with NAFLD cells or HepG2 cells (number of cells: 1×10^6^ cells) in 1 ml of binding buffer (BB) at 4°C for 1 h, stained with DAPI, and finally washed with washing buffer three times, and immunofluorescence images were captured.

##### 2.2.2.2 Flow cytometry

The experimental groupings were the same as that described in Section 2.2.2.1. The methods were as follows. All cells (5×10^5^) were incubated with NAFLD01 labeled with FITC at 250 nM and BB at 200 μl at 4°C for 1 h. FITC-labeled nonsense sequences were used as controls. After incubation, the unbound adapter was washed away, and the cells were suspended in 500 μl WB. An Attune NxT flow cytometer was used to measure the fluorescence intensity.

#### 2.2.3 Synthesis and characterization of aptamer-modified ultrasonic nanobubbles loaded with resveratrol and ultrasmall copper-based nanoparticles

##### 2.2.3.1 Synthesis of Cu_5.4_O NPS

CuCl_2_ (10 mM) was dissolved in 50 ml of deionized water and stirred magnetically for 10 min in an 80°C oil bath. Then, l-ascorbic acid (100 mM, 50 ml) was slowly added. After that, the pH was adjusted to 8.0–9.0 with sodium hydroxide, and the mixture was stirred at 80°C for 12 h. At the end of the reaction, the large aggregated particles were removed by centrifugation (6,577 × g, 15 min), and the supernatant was dialyzed for 2 days (molecular weight: 10,000 Da) to remove small molecules. Purification of Cu_5.4_O NPs was concentrated by centrifugation.

##### 2.2.3.2 Synthesis of ultrasonic nanobubbles

DPPC and DSPE-PEG (2000) were dissolved in 2 ml chloroform at a mass ratio of 10:4. The mixture was transferred to a 25 ml glass flask with a rotating evaporator. A dry lipid film was produced in a commercially available rotary evaporator at 55°C and 130 RPM for 10 min by rotary evaporation. The phospholipid mixture was dried in 1.5 ml of hydrated solution (10% glycerol and 90% 1 × PBS, V/V). Then, the flask was placed in a culture shaker at 37°C and 120 RPM for 60 min to prepare the liposome membrane suspension. Subsequently, the liposome suspension was placed in a vial sealed with a rubber cap. A 50-ml syringe with a long needle was used to extract the gas in the vial, and then C_3_F_8_ gas (the bubble core was formed later) was added to the vial until the pressure in the vial was balanced. Finally, the vials were placed in a mechanical vibrator and subjected to mechanical vibration for 90 s. The stock suspension of ultrasonic nanobubbles was immediately placed on ice. The whole process was performed in the absence of light.

##### 2.2.3.3 Aptamer-functionalized nanobubbles

**SCHEME 1:**
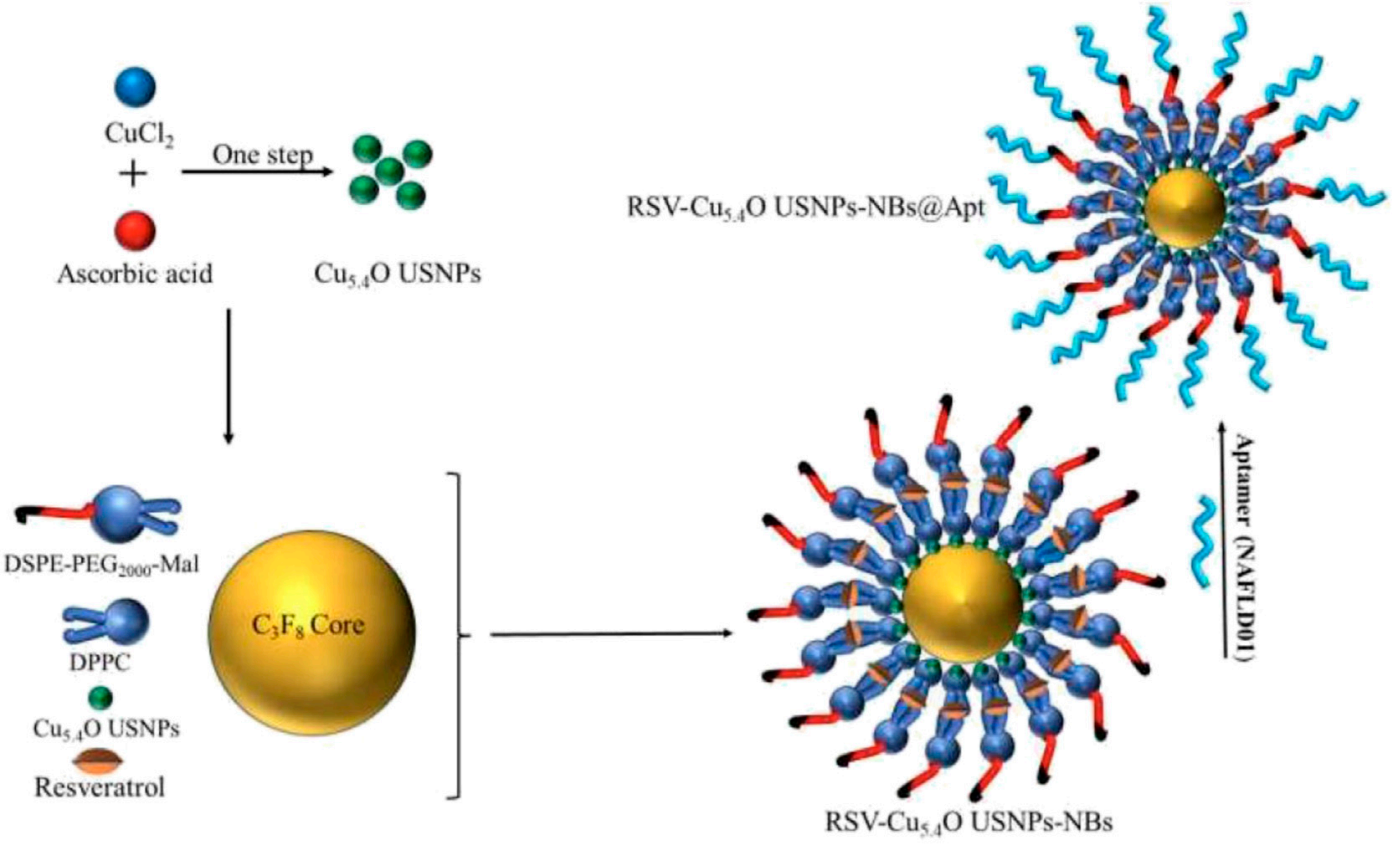
Schematic diagram of the preparation of Apt-NBs@Res@Cu_5.4_O NPs.

1) DSPE-PEG (2000) was replaced with DSPE-PEG (2000)-Mal during the synthesis of the nanobubbles. 2) Next, 10 mM EDTA, 10 mM Tris (2-carboxyethyl) phosphine hydrochloride and 2 mM NAFLD01 aptamer were reacted at 37°C for 1 h, and then nanobubbles were added and incubated for 2 h. Then, the aptamer-modified nanobubbles floating in the upper layer of the suspension were collected by washing and centrifugation with cold PBS solution three times (300 RPM, 3 min). 3) The NBs@Res/Apt-NBs@Res preparation process was the same as that described for the nanobubbles/Apt-NBs, and Res was added at to a mass ratio of 10:4:0.3. 4) The NBs@Cu_5.4_O NPs/Apt-NBs@Cu_5.4_O preparation process was the same as that described for the nanobubble/Apt-NBs, with Cu_5.4_O addition at a 10:4:0.3 mass ratio. 5) The NBs@Res@Cu_5.4_O NPs/Apt-NBs@Res@Cu_5.4_O preparation process was the same as that described for nanobubbles/Apt-NBs; the mass ratio was set to 10:4:0.3:0.3, and Res (1 mg/ml) and Cu_5.4_O NPs were added at the same time (1 mg/ml, PBS). The preparation process of Apt-NBs@Res@Cu_5.4_O NPs is shown in Scheme 1. 6) Transmission electron microscopy (TEM), XRD, X-ray photoelectron spectroscopy (XPS), UV–VIS spectroscopy, energy-dispersive X-ray spectroscopy (EDS) and other approaches were used for characterization.

#### 2.2.4 Aptamer-modified ultrasonic nanobubbles loaded with resveratrol and ultrasmall copper-based nanoparticles can be used for effective treatment of nonalcoholic fatty liver disease

##### 2.2.4.1 CCK8 analysis of the of Apt-NBs@Res@Cu_5.4_O NPs

HepG2 cells (1×10^3^) were inoculated into a 96-well plate and cultured in DMEM complete medium containing 620, 310, 155 or 77.5 nM N-bromosuccinimide (NBS) (the initial concentration of NBS was 620 nM) for 48 h. Then, the cells were washed with PBS 3 times and incubated with 10 μl CCK-8 reagent (Bimake) for 2 h. Finally, the cell viability was measured with a microplate reader (BioTek) at an absorption wavelength of 450 nm. The experiment was repeated 3 times, and care was taken to avoid light during the experiment.

##### 2.2.4.2 Aptamer-modified ultrasonic nanoparticles loaded with resveratrol and ultrasmall copper-based nanoparticles for the treatment of nonalcoholic fatty liver

The experimental groups were as follows: 1) HepG2 cells + ultrasound; 2) HepG2 cells + high oil fatty acid + ultrasound; 3) HepG2 cells + NBs + high-oil fatty acid + ultrasound; 4) HepG2 cells + NBs@Res@Cu_5.4_ONPs + high-oil fatty acid + ultrasound; 5) HepG2 cells + Apt-NBs@Res@Cu_5.4_ONPs + ultrasound. The HepG2 cells were placed in a 6-well plate, and high-oil fatty acidscontaining different nanobubbles were added (the maximum concentration of the nanobubbles without notable cytotoxicity was selected as the concentration for administration), and after incubation for 24 h, ultrasound irradiation (WED-100 ultrasound) was performed. After that, the cells were washed with cold PBS and collected. The expression of TNF-α and IL-10 was detected by Western blotting, and the level of total triglycerides (TGs) was determined by ELISA. The concentration of Res in each group was 90.6 mM (initial concentration), and the experiment was performed in triplicate.

### 2.3 Statistical analysis

SPSS 22.0 was used for statistical analysis. All data are expressed as mean ± SD, and one-way ANOVA was used for comparisons among groups. The LSD method was used for homogeneity of variance, and the Welch method or Brown⁃Forsythe method was used for correction of homogeneity of variance. Dunnett’s T3 method was used for comparisons between groups. *p* < 0.05 indicated a statistically significant difference.

## 3 Results and discussion

NAFLD is associated with the production of ROS and oxidative stress (Schwimmer, 2007; Lomonaco et al., 2013; Braud et al., 2017; Kasper et al., 2021; Ding et al., 2022). The clinical use of broad-spectrum antioxidants such as N-acetylcysteine and acetyl-L-carnitine, which are currently used to clear ROS, is limited due to their poor bioavailability, low stability and low efficacy. One promising strategy is to develop nanozymes to maintain the natural redox balance in biological systems. CuNPs have a wide range of enzyme-like activities, the ability to clear ROS and ameliorate ROS-related diseases, excellent biocompatibility and high renal clearance. They have been shown to be effective in the treatment of a wide range of ROS-related diseases and to have no significant toxic effects (Liu et al., 2020). In addition, CuNPs are currently a research hotspot because they are easy to combine with other polymers and show better stability than other nanoparticle polymers, with a large surface area and a unique morphology. Res has been widely studied by researchers because of its inhibitory effect on oxidative stress. To solve the problem of Res’s poor water solubility, instability and ease of metabolism in the intestinal tract, Wan et al. (2018) developed PLGA nanoparticle-based RSV-PLGA-NPs that can be loaded with Res for successful treatment of NAFLD. The authors concluded that RSV-PLGA NPs can release RSV continuously and have high stability, water solubility and nontargeting properties. Teng et al. (2019) used liver-targeted oxidized starch lysozyme (OSL) as a nanocarrier to target the liver with covalently coupled galactose (Gal), which can be recognized by specifically expressed salivary glycoprotein receptors in liver cells. The nanocarrier specifically delivers Res to the liver to increase its therapeutic effectiveness. Although ultrasound nanobubbles are more suitable for delivering drugs than the abovementioned nanocarriers because of their ability to enter extravascular tissue, support specific ultrasound imaging, and achieve targeted drug release, it is necessary to modify them with specific targeting groups to improve their therapeutic efficacy. In view of the high affinity and selectivity of aptamers, as well as their advantages of good stability, easy chemical modification and coupling, and low cost, we used aptamer-functionalized ultrasound nanobubbles as nanoparticle carriers to design and synthesize particles loaded with Res and CuNPs for synergistic treatment of NAFLD.

### 3.1 Establishment of a nonalcoholic fatty liver disease cell model induced by high-oil fatty acids

**FIGURE 1:**
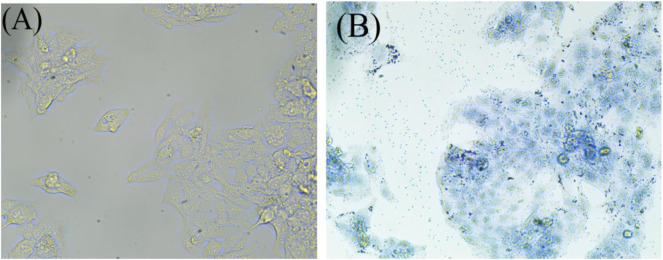
Oil red before staining **(A)**, and Oil red after staining **(B)**. The NAFLD cell model was established by oil red O staining. After being stained with oil red O and observed under a microscope with magnification of ×40, obvious lipid droplets were formed.

As shown in Figure 1, HepG2 cells were treated with high-oil fatty acids (palmitic acid/oleic acid ratio of 1/2, final concentration of 4 mM), and many lipid droplets formed after 48 h, which confirmed the successful establishment of the NAFLD cell model.

### 3.2 Ability of the aptamer to target nonalcoholic fatty liver cells

There is a need to develop new effective methods and therapies for NAFLD, such as molecular probes that specifically recognize NAFLD cells. Nucleic acid aptamers (Zhou and Rossi, 2017), single-chain oligonucleotide molecules, have the advantages of a low molecular weight, no immunogenicity, a low synthesis cost and high stability after chemical modification. Aptamers can selectively bind to target sites through their unique three-dimensional structures and have a high affinity and a specificity similar to that of antigen-antibody interactions. Therefore, aptamers have been widely used in diagnosis, screening and drug development. Some aptamers not only recognize disease target molecules but also can be modified as drugs. Pu et al. (2021) developed a NAFLD cell-specific aptamer called NAFLD01 that specifically recognizes cultured steatotic hepatocytes and fatty liver tissue slices. It also improved the fatty acid degradation of NAFLD cells and increased the expression of PPAR-α (peroxisome activating receptor-α). Therefore, in this study, liver targeting was achieved by modifying the NAFLD01 aptamer. In our study, the NAFLD01 aptamer was used to modify ultrasound nanobubbles, and the specific binding ability of the NAFLD01 aptamer to NAFLD cells was verified through multiple experiments.

#### 3.2.1 Confocal fluorescence microscopy imaging demonstrated specific binding of the NAFLD01 aptamer to nonalcoholic fatty liver disease cells

**FIGURE 2:**
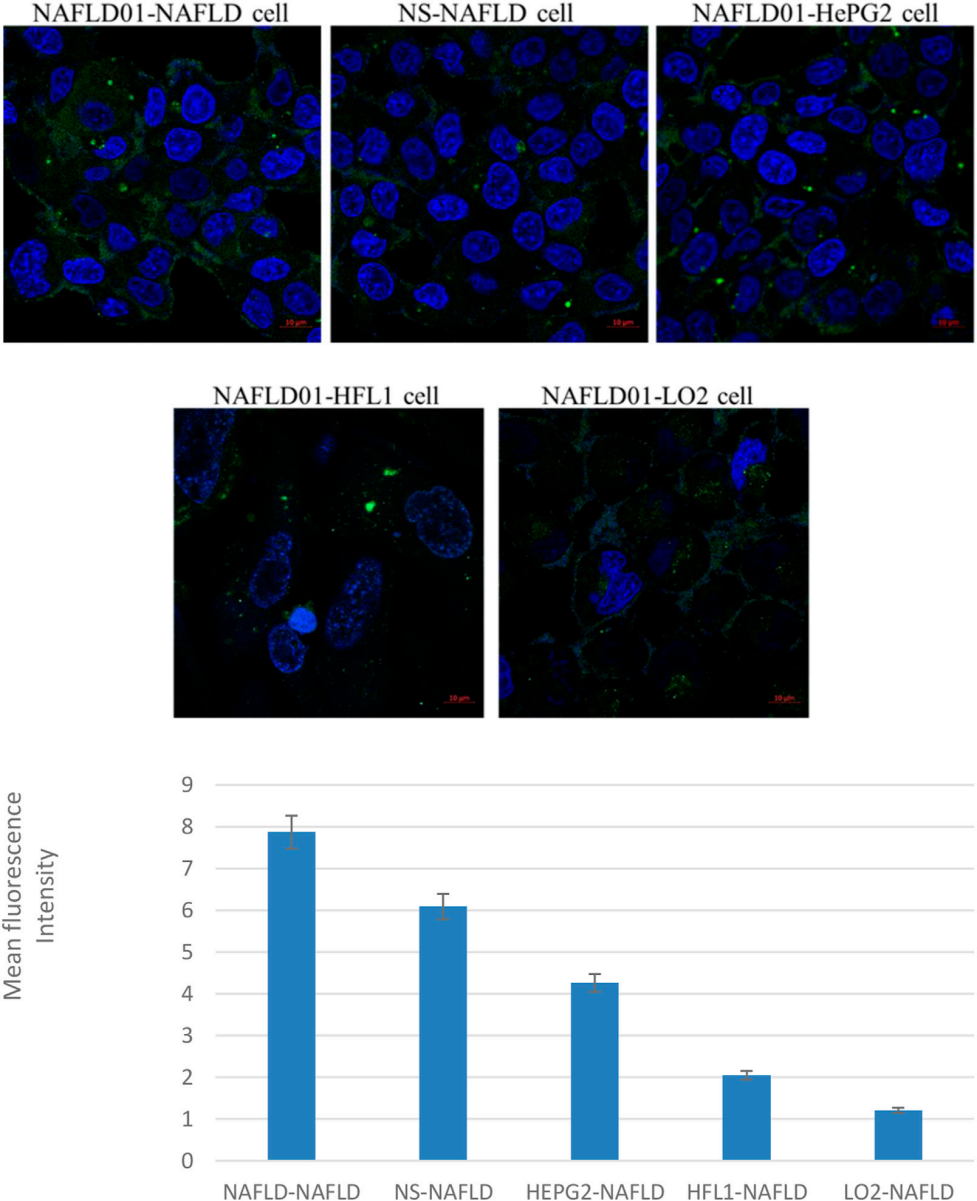
Confocal fluorescence verifies that aptamers can target NAFLD cells. Compared to HePG2 cells, LO2 cells, and HFL1 cells, NAFLD cells showed stronger green fluorescence signals. In addition, stronger green fluorescent signals were also shown around NAFLD01-NAFLD cells compared to NS-NAFLD cells. Quantification analysis of fluorescence intensity of the five groups (*p < 0.05).

As shown in Figure 2, the fluorescence intensity of the fluorescein-labeled aptamer showed that NAFLD01 exhibited the strongest binding ability to NAFLD cells compared to nonsense sequences or other cells, which demonstrates that the NAFLD01 aptamer binds specifically to NAFLD cells and has potential for targeted treatment of NAFLD.

#### 3.2.2 Flow cytometry verified the specific binding of the NAFLD01 aptamer to nonalcoholic fatty liver disease cells

**FIGURE 3:**
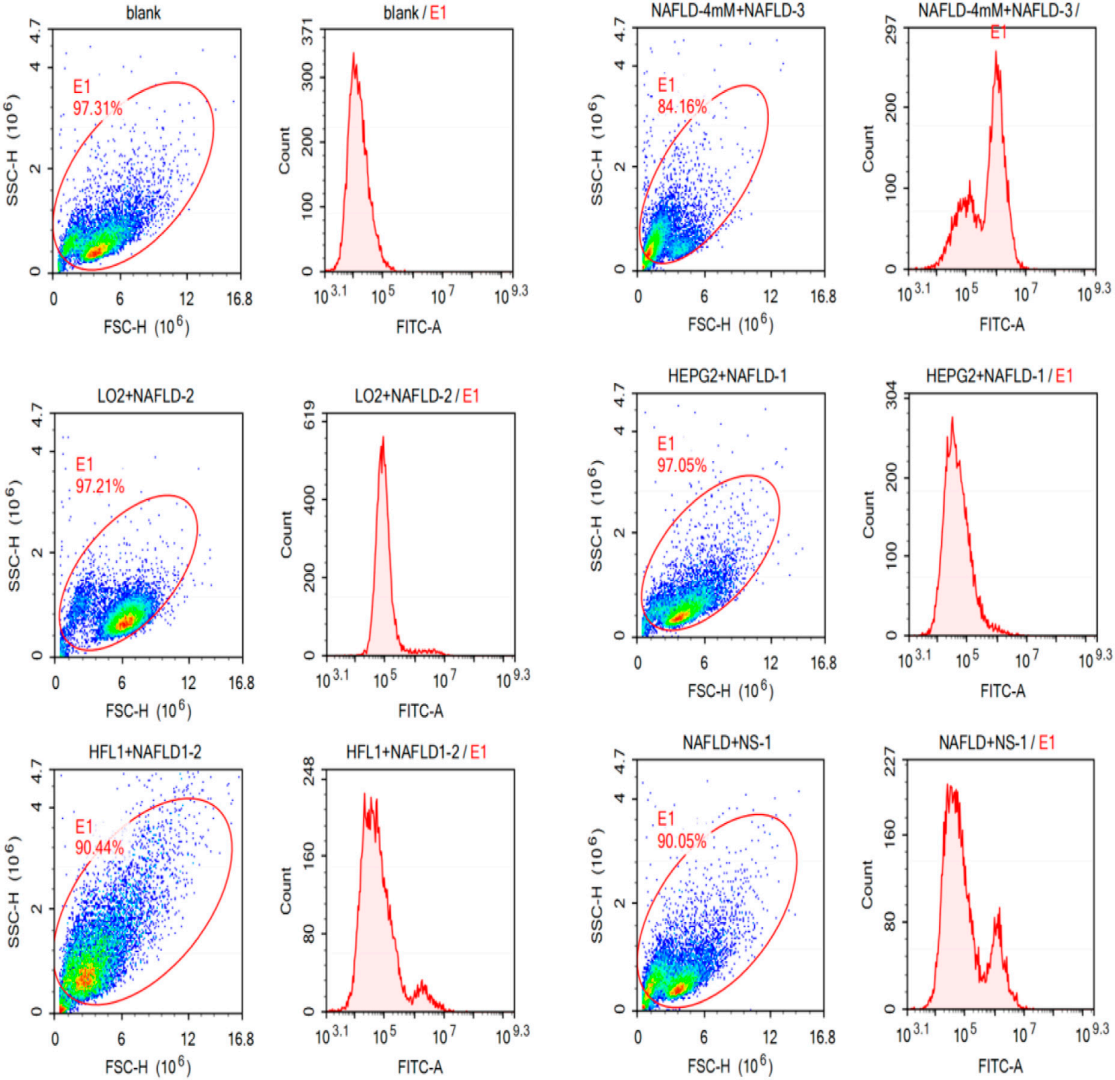
NAFLD01 aptamer’s specific binding to NAFLD was verified by flow cytometry. The NAFLD01 aptamer sequence exhibited stronger fluorescence on NAFLD cells than the antisense aptamer sequence or other LO2, HFL1, and HepG2 cells.

As shown in Figure 3, the NAFLD01 aptamer sequence exhibited stronger fluorescence on NAFLD cells than the antisense aptamer sequence or other LO2, HFL1, and HepG2 cells. The fluorescence signal intensity decreased in the order NAFLD cells > NS-1 cells > HFL1 cells > HepG2 cells > LO2 cells > blank group, which further proved that the NAFLD01 aptamer binds specifically to NAFLD cells and has potential for targeted treatment of NAFLD.

#### 3.2.3 Synthesis and characterization of aptamer-functionalized ultrasound nanobubbles loaded with Res and CuNPs

**FIGURE 4:**
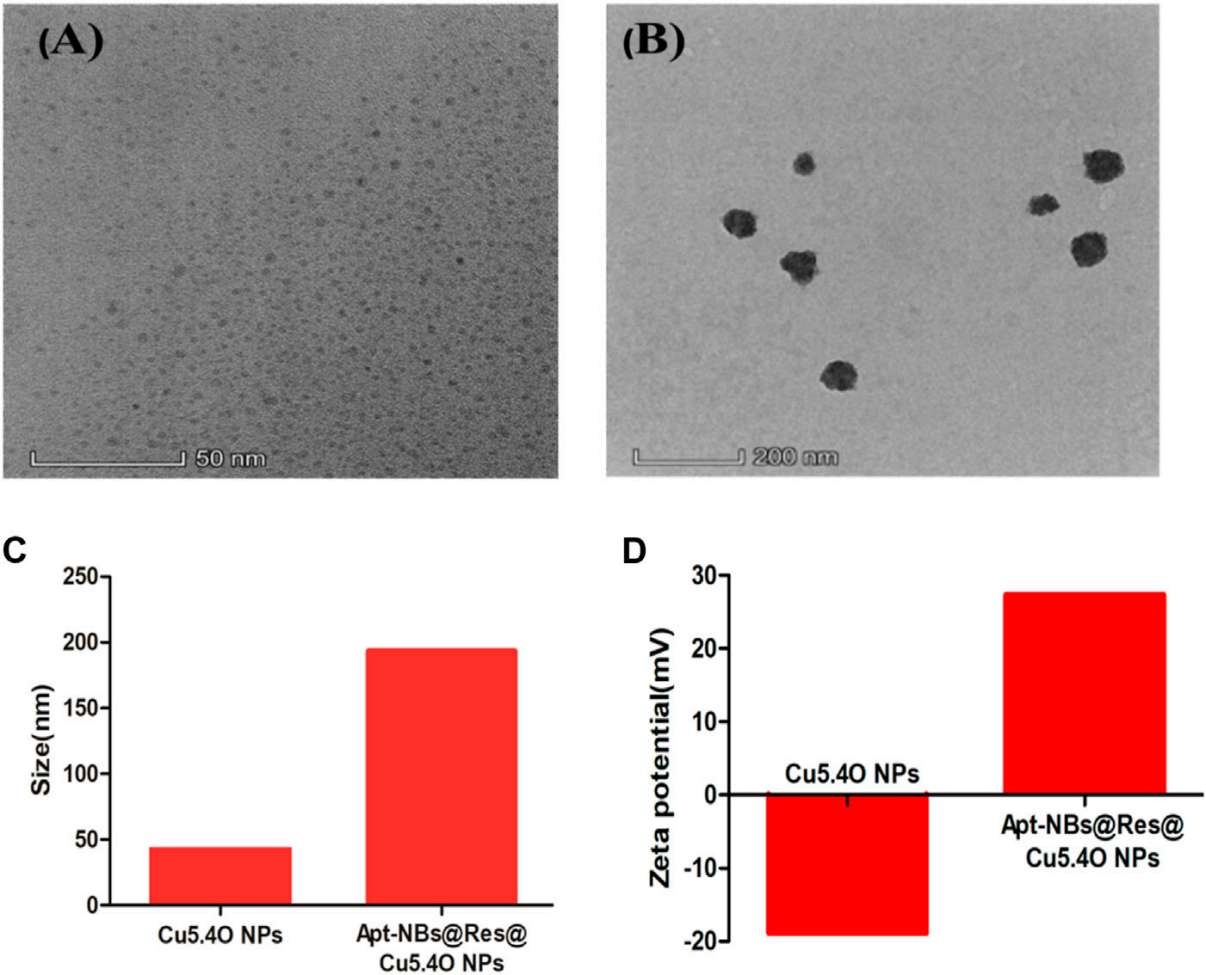
The TEM spectra of Cu_5.4_ONPs **(A)**, and Apt-NBs@Res@Cu_5.4_ONPs **(B)**; the size **(C)** and Zeta **(D)** of Cu_5.4_ONPs and Apt-NBs@Res@Cu_5.4_ONPs.

**FIGURE 5:**
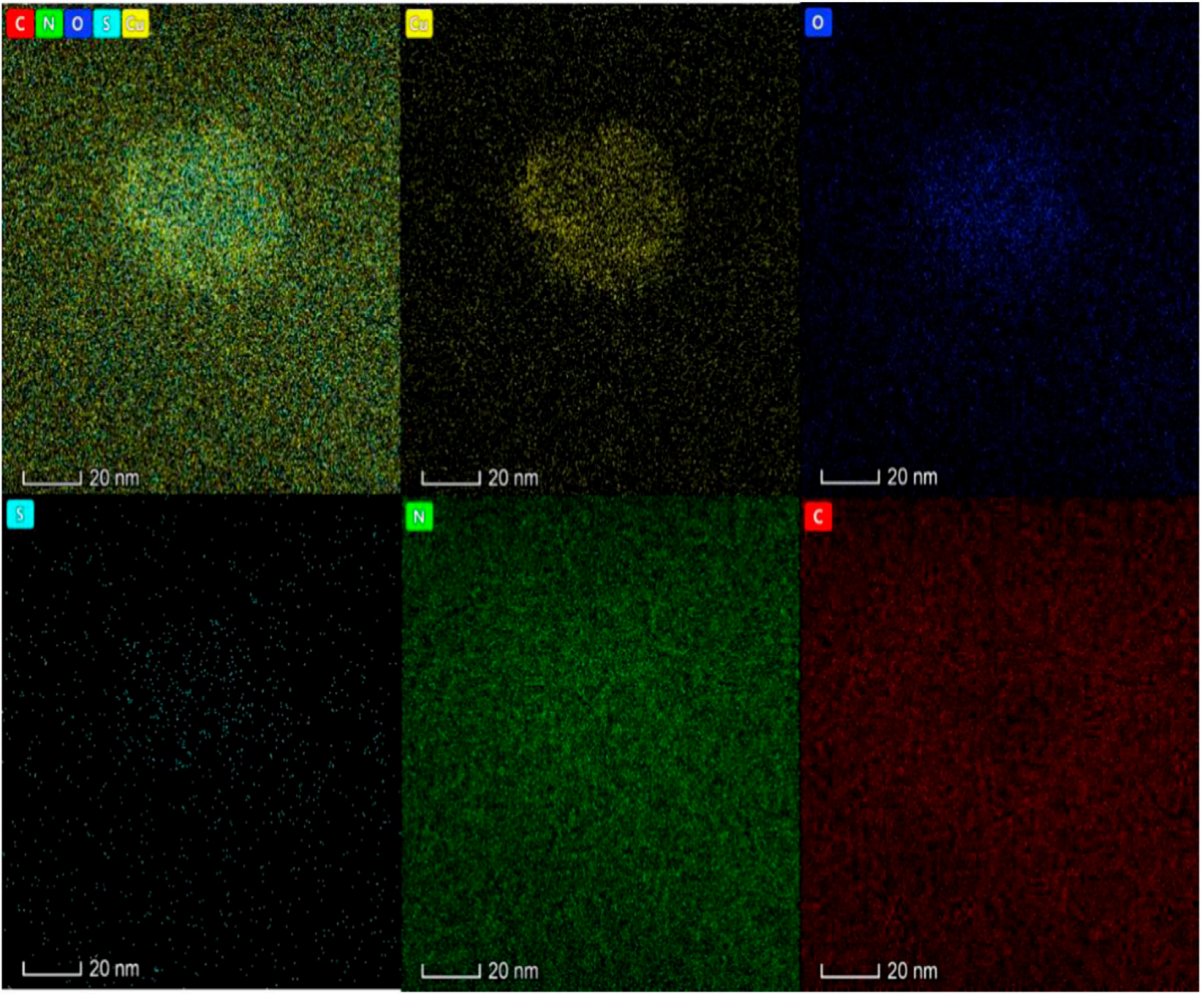
The Energy dispersive spectra of Apt-NBs@Res@Cu_5.4_ONPs shows that the material also has five elements: Cu, N, O, S and C, in which Cu is mainly located in the core of the material and N is distributed in the shell.

**FIGURE 6:**
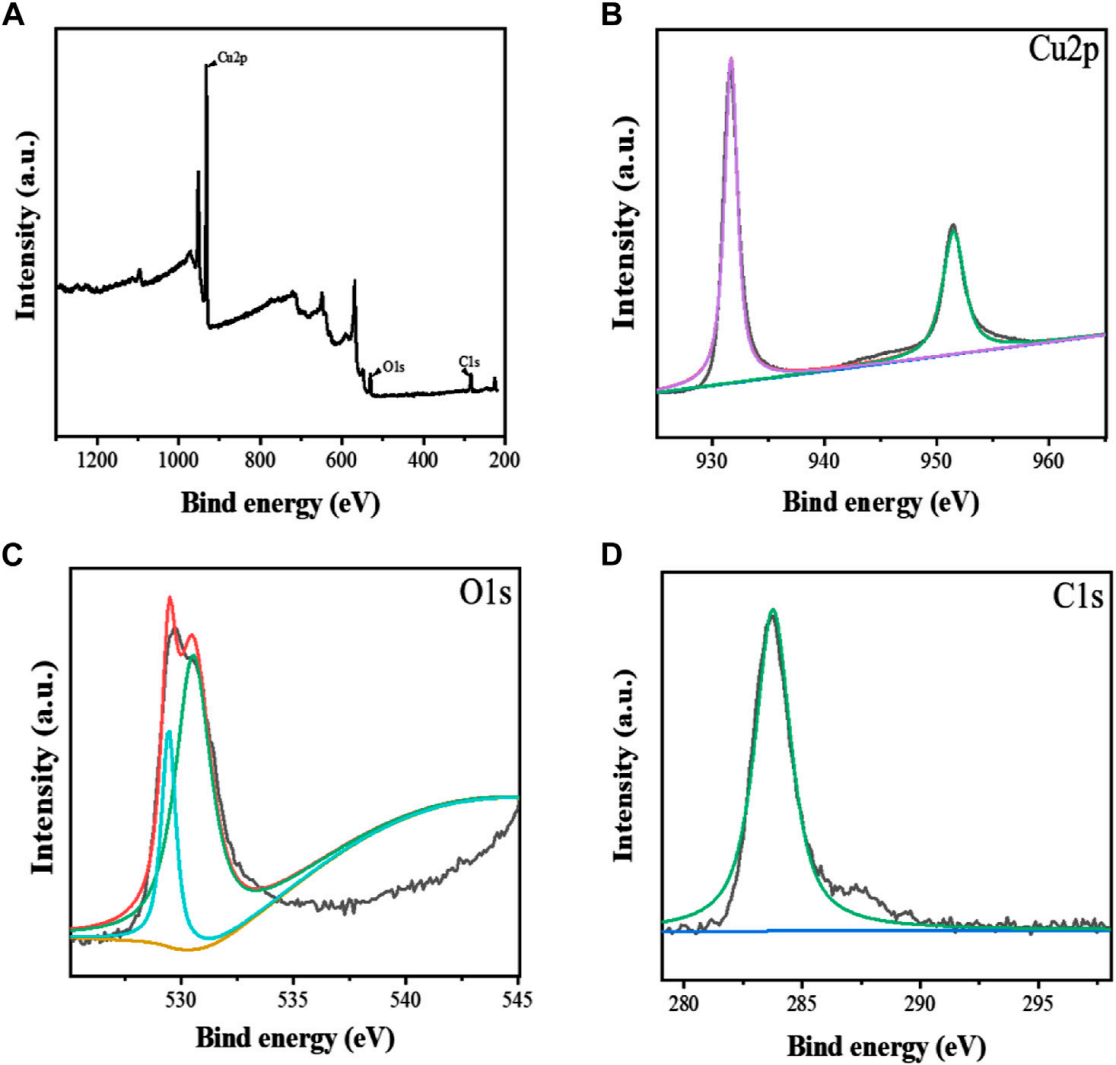
The XPS spectra of Apt-NBs@Res@Cu_5.4_O NPs **(A)**, The Cu2p **(B)**, O1s **(C)**, C1s **(D)** spectra of Apt-NBs@Res@Cu_5.4_ONPs.

**FIGURE 7:**
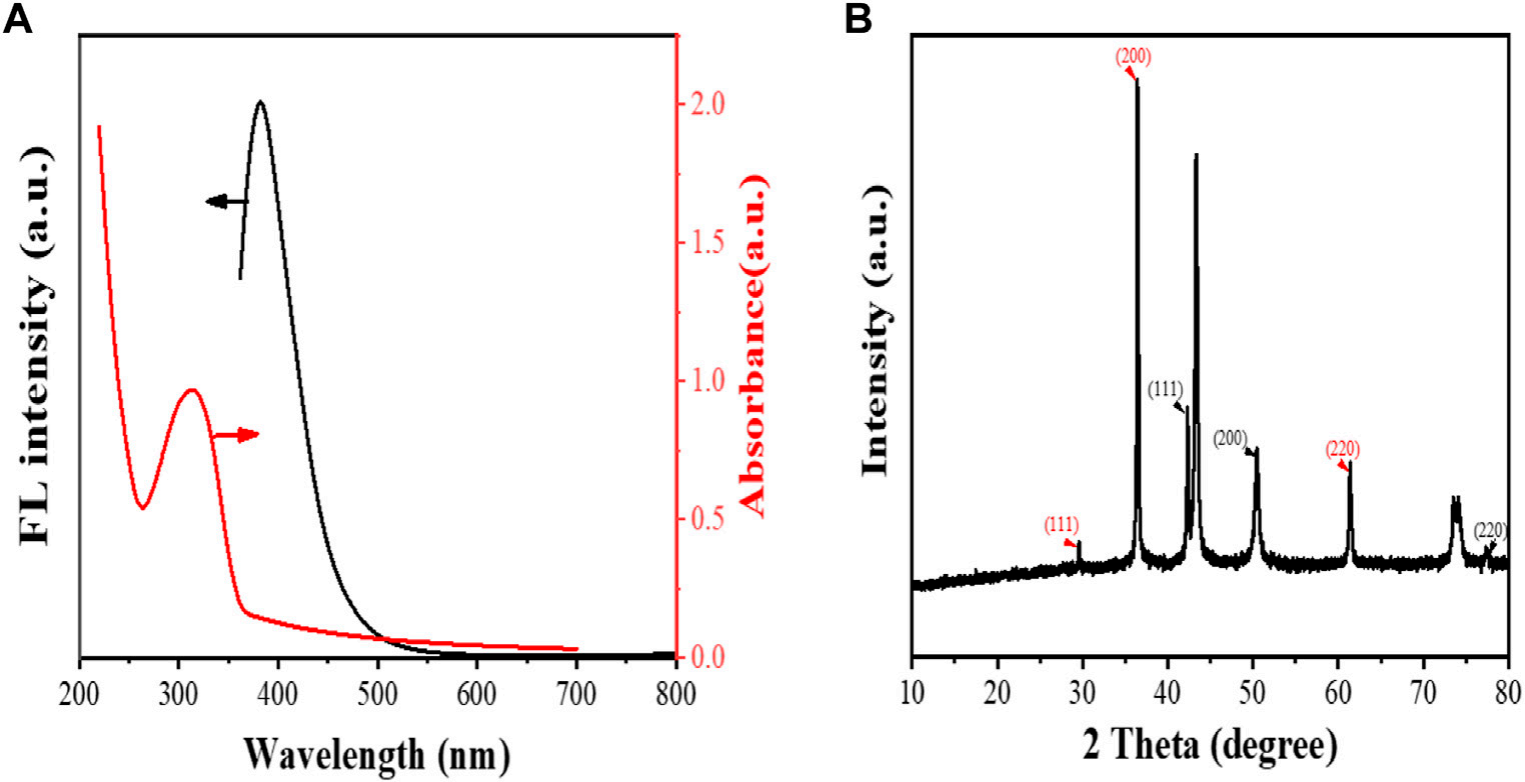
The fluorescence and UV-Vis spectra **(A)**, and XRD spectra of Apt-NBs@Res@Cu_5.4_O NPs **(B)**.

**FIGURE 8:**
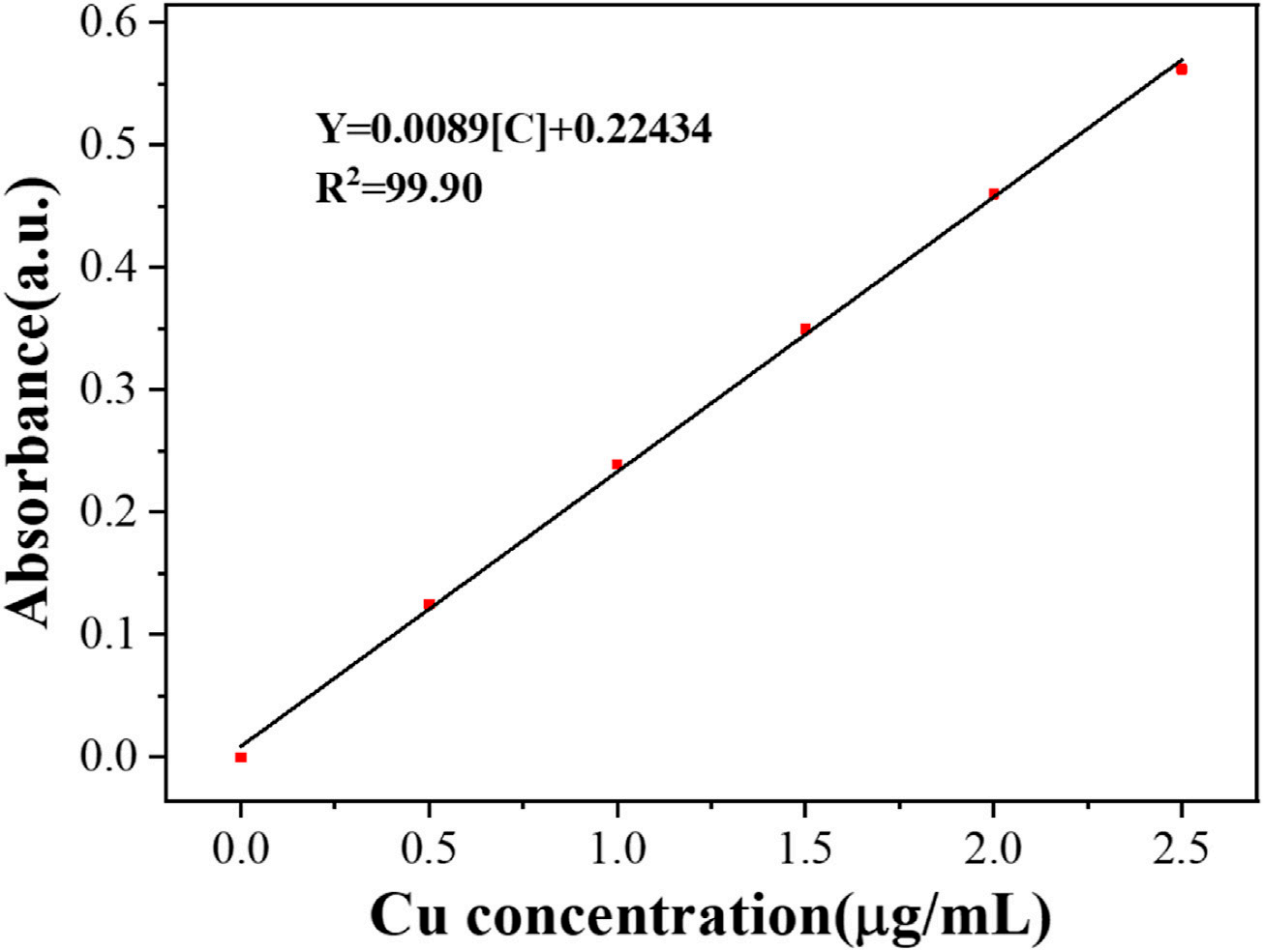
The linear calibration plot for Cu concentration by using Atomic Absorption Spectroscopy.

**FIGURE 9:**
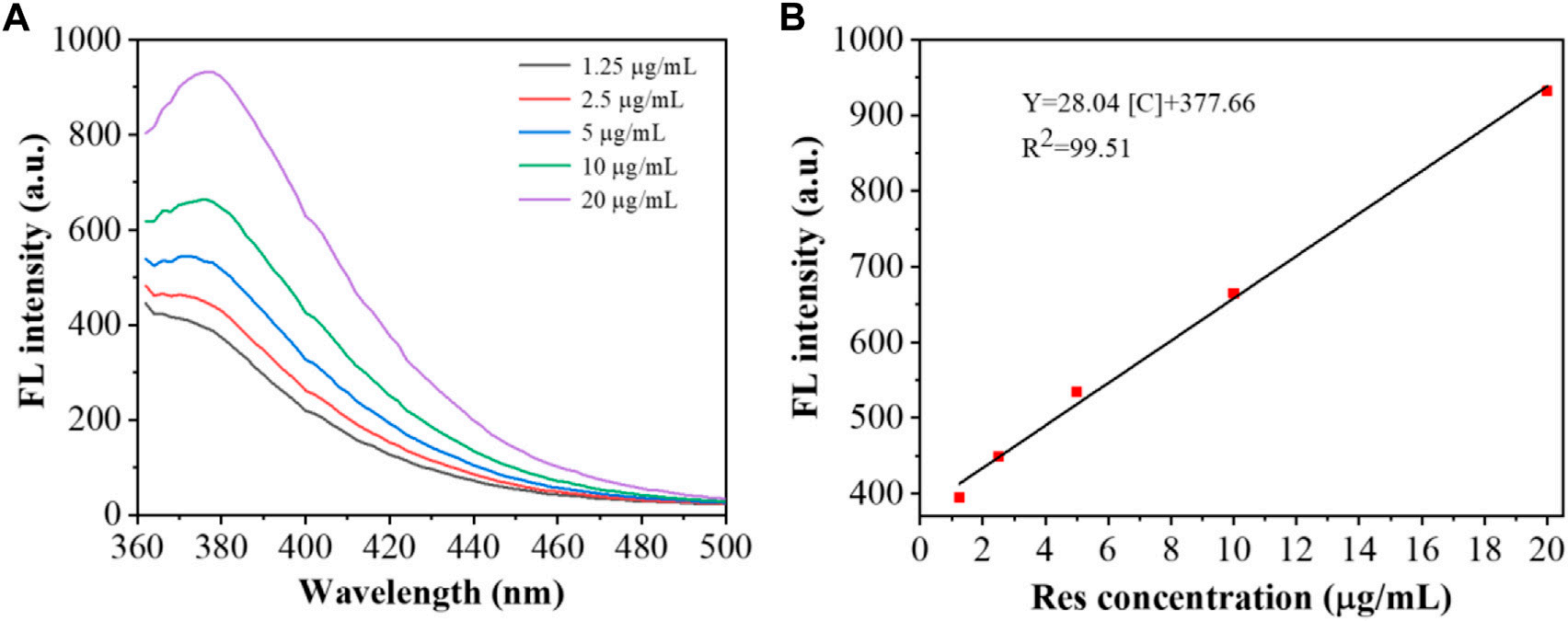
Fluorescence spectra **(A)** and corresponding linear calibration plot for Res **(B)**.

Zhu et al. (2018) constructed aptamer-modified nanobubbles for ultrasound imaging of various tumor cells. To date, there has been no research on the construction of NAFLD aptamer-modified nanobubbles or the synthesis of nanobubbles loaded with Res and CuNPs. We synthesized a nanobubble by thin-film hydration and mechanical oscillation, which is a relatively simple, green and economical method. As shown in Figures 4, 5, the morphology and size potential distribution of the materials were characterized by TEM and a Malvern particle size potentiometer. CuNPs have a small and uniform particle size distribution, and their particle size and zeta potential were 46.13 nm and −18.9 mV, respectively. When Res and CuNPs were loaded into the nanobubbles, the particle size and zeta potential of the nanobubbles changed to 127.6 nm and 35.73 mV, respectively. Then, the aptamer with NAFLD was covalently modified with maleamide and sulfhydryl groups, and the size and zeta potential of the nanobubbles changed to 193.73 nm and 27.39 mV, respectively. EDS, XRD, and XPS spectra of ultrasonic nanobubbles were also obtained. The results are shown in Figures 6–9 and indicate that the final synthesized aptamer-functionalized nanobubbles contained Cu, N, C, O, and S, among which Cu was mainly distributed in the inner position of the nanobubbles due to its hydrophilic properties. The C, N, and O from Res are mainly distributed in the middle of nanobubbles due to their hydrophobic properties. Encapsulation of Res in PLGA as a carrier can enhance its stability, solubility and pharmacological potential. The loading capacity of Res was measured using XPS and XRD. The results showed that the loading capacity of Res was 206.8 mg, and the loading rate was 94%, while the loading capacity of CuNPs was 6 mg (10 ml). We prepared nanoparticles with an encapsulation efficiency of up to 90% or higher, which showed excellent drug loading ability. Zeta potential is an important indicator of electrode stability. Colloidal dispersions can resist aggregation. The higher the zeta potential is (positive or negative), the more stable the system. As the absolute zeta potential is greater than the electrostatic repulsion, the nanobubble potential was 27.39 mV, which indicated that apt-NBs@Res@Cu_5.4_O NPs have high colloidal stability (Chen et al., 2016; Carbone et al., 2018). All of the above data effectively proved the successful synthesis of Res and CuNPs supported by adaptor-functionalized ultrasound nanobubbles.

### 3.3 Aptamer-modified ultrasound nanobubbles loaded with Res and CuNPs could be used to treat nonalcoholic fatty liver disease effectively

#### 3.3.1 *In vitro* cytotoxicity test

**FIGURE 10:**
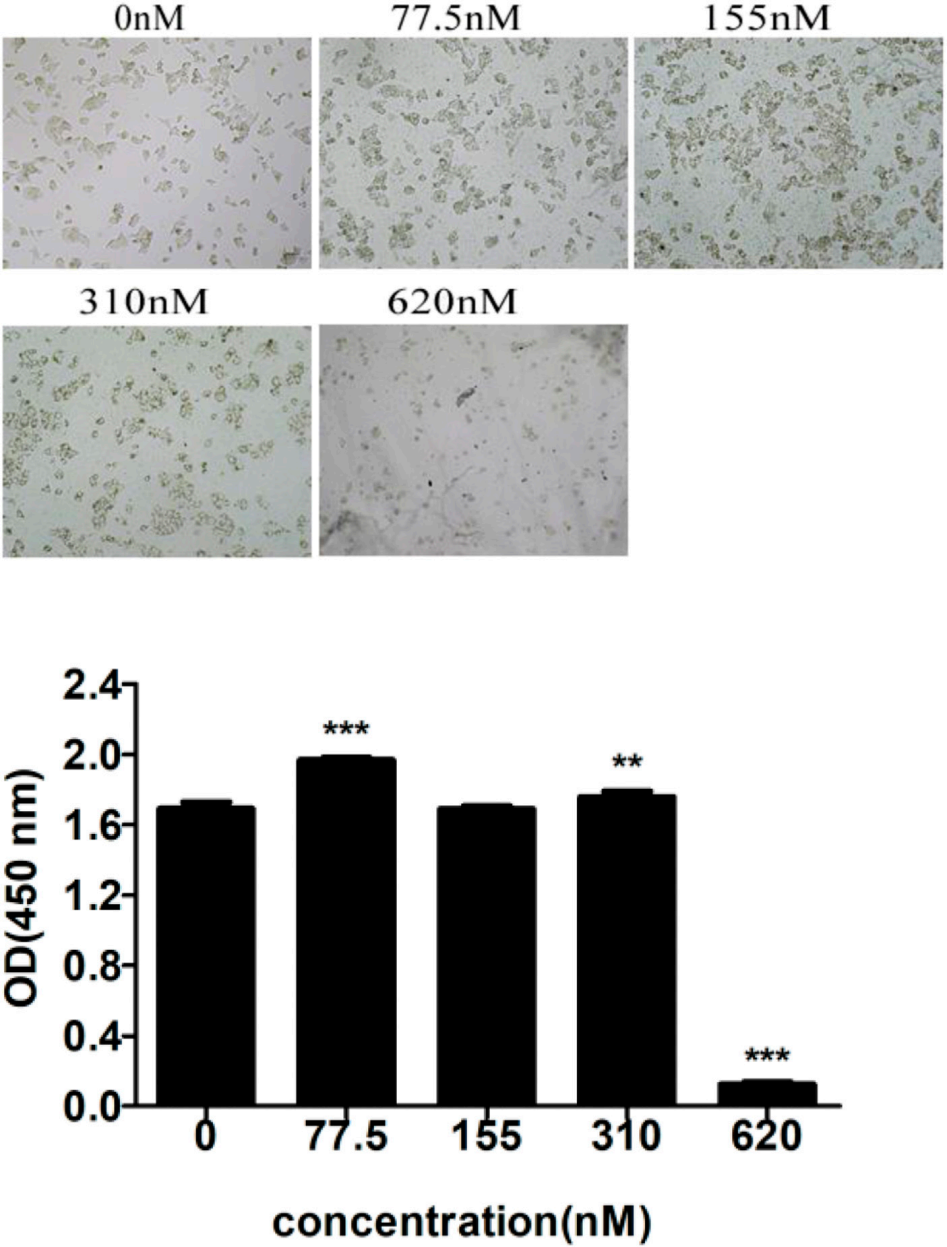
Cytotoxicity analysis of Apt-NBs@Res@Cu_5.4_ONPs by CCK8.

Cytotoxicity analysis of Apt-NBs@Res@Cu_5.4_O NPs with CCK8. As shown in Figure 10, nanobubbles no larger than 310 nM (Apt as the measurement unit) were basically not cytotoxic, and this concentration was used for subsequent treatment of NAFLD.

#### 3.3.2 The expression levels of the inflammatory factors TNF-α and Il-10 were detected by Western blotting, and the level of total TGs was detected by ELISA

**FIGURE 11:**
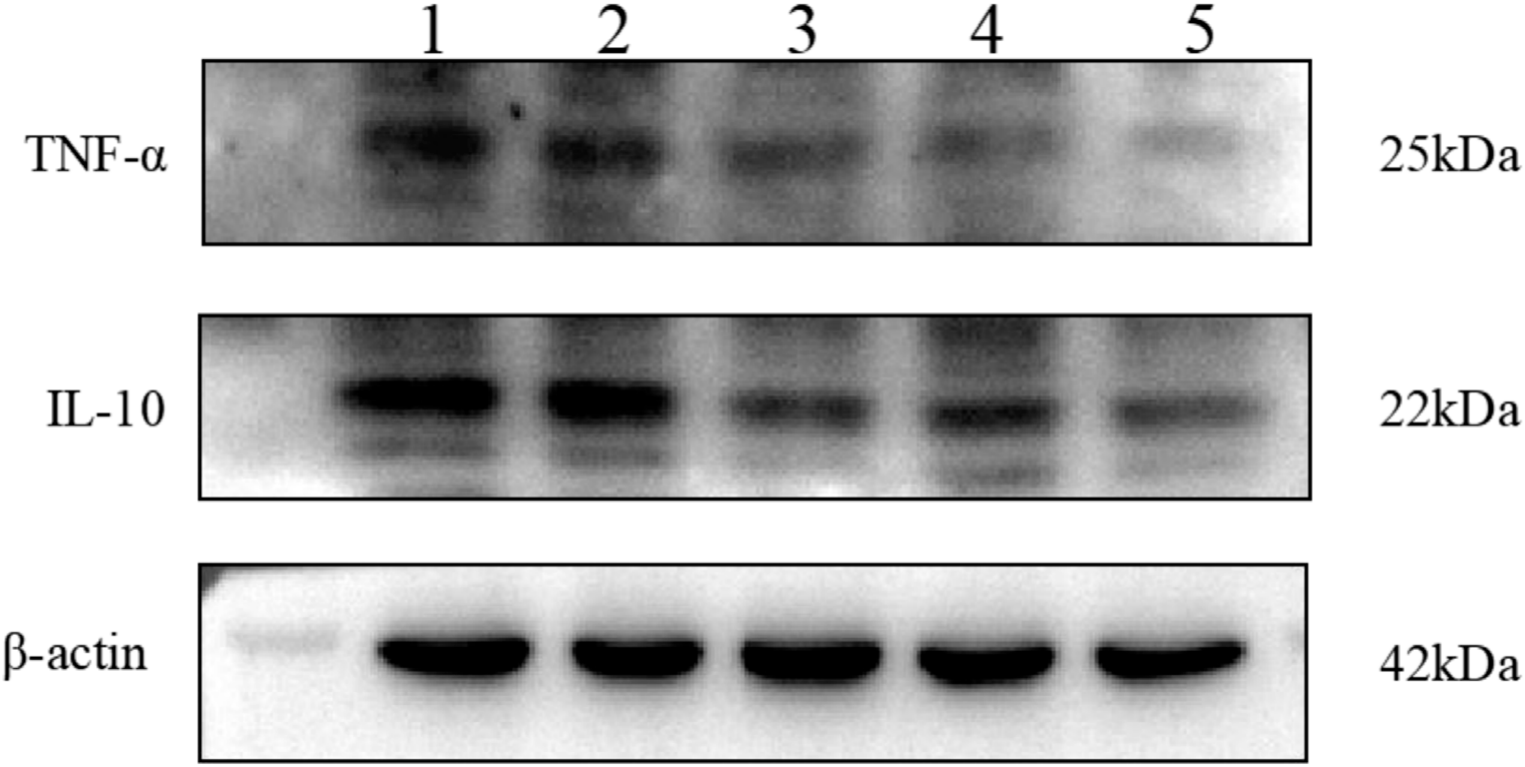
The effect of different treatment on IL-10 and TNF-α expression of HepG2 cell. 1) ultrasound; 2) FFA + ultrasound; 3) NBs + FFA + ultrasound; 4) NBs@Cu_5.4_O NPs@Res + FFA + ultrasound; 5)Apt-NBs@Cu_5.4_ONPs@Res + FFA + ultrasound.

**FIGURE 12:**
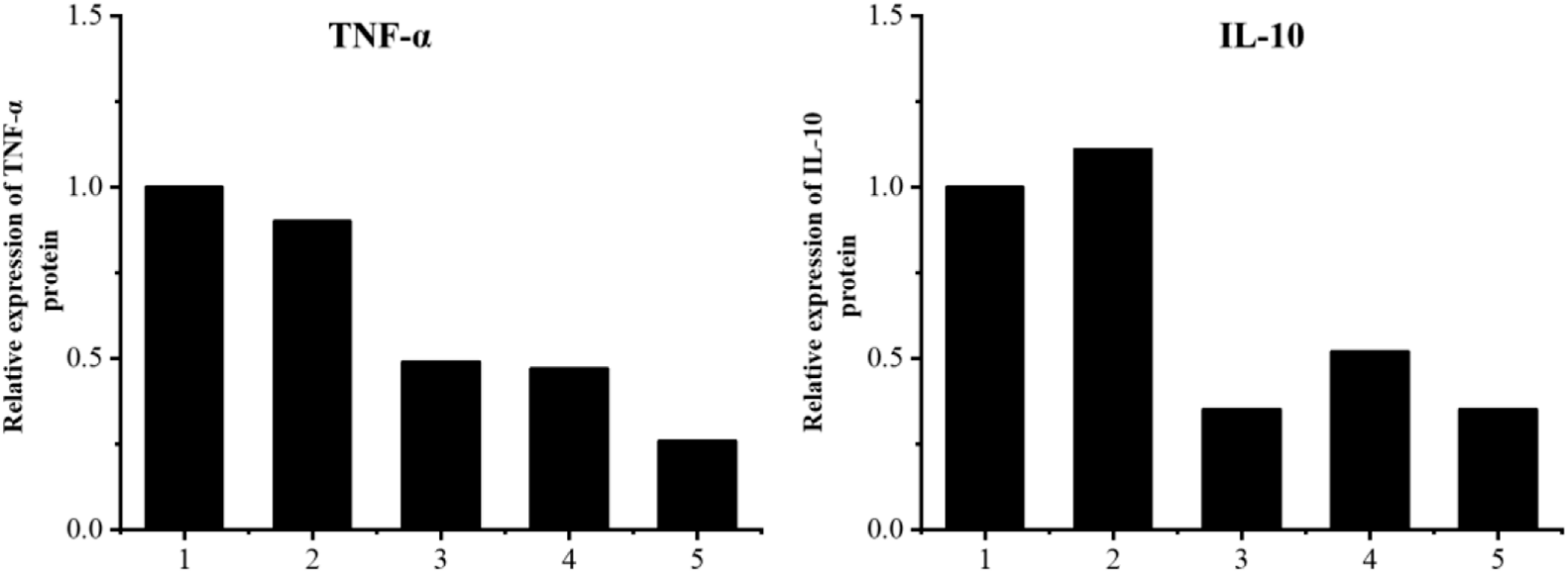
Relative levels of TNF-α and IL-10 expression after indicated treatments. 1) ultrasound; 2) FFA + ultrasound; 3) NBs + FFA + ultrasound; 4) NBs@Cu_5.4_ONPs@Res + FFA + ultrasound; 5) Apt-NBs@Cu_5.4_ONPs@Res + FFA + ultrasound.

**FIGURE 13:**
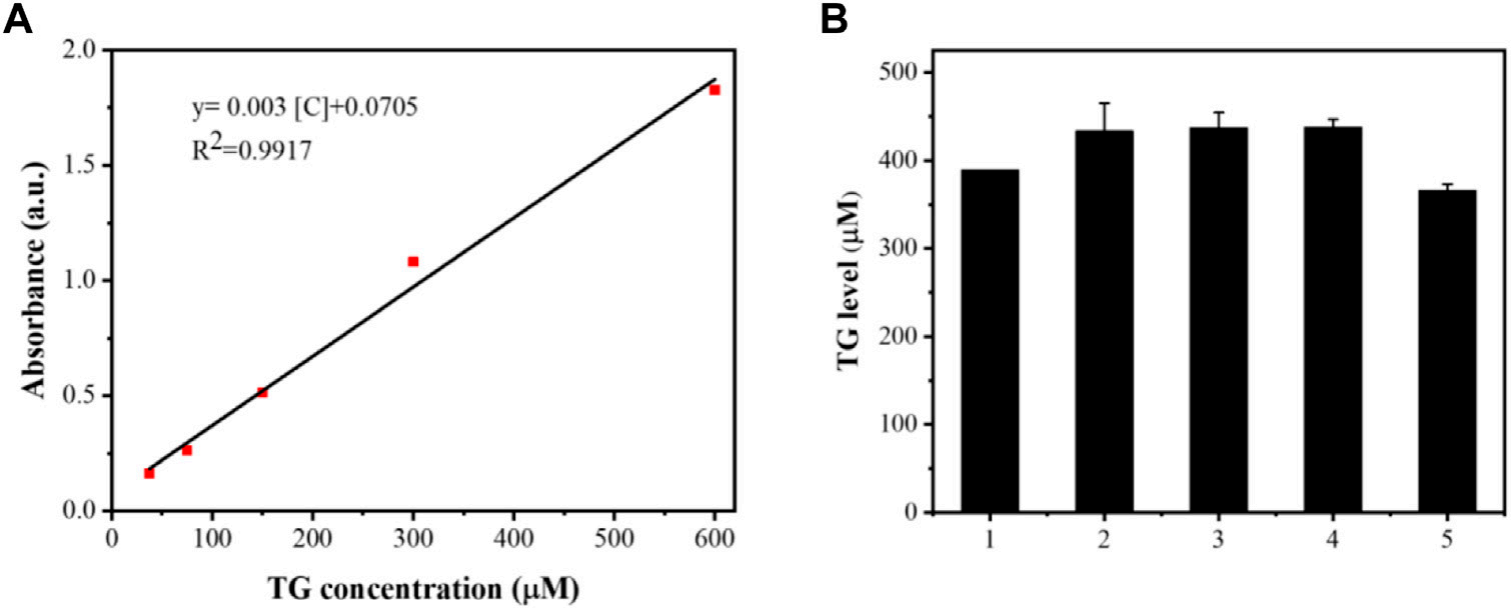
The linear calibration plot for TG concentration by ELISA method **(A)** and TG level after different treatment **(B)**; 1) ultrasound; 2) FFA + ultrasound; 3) The NBs + FFA + ultrasound; 4) NBs@Cu_5.4_ONPs@Res + FFA + ultrasound; 5) Apt-NBs@Cu_5.4_ONPs@Res + FFA + ultrasound.

The results are shown in Figures 11–13. Compared with that in the other groups, the expression of the inflammatory factors TNF-α and IL-10 in the HepG2 + Apt-NBs@Cu_5.4_O NPs@Res + FFA + ultrasound group was the lowest, while the level of total TGs in the HepG2 + Apt-NBs@Cu_5.4_O NPs@Res + FFA + ultrasound group was lower than that in the FFA + ultrasound group, and there was no significant difference between them.

NASH, an inflammatory phase of NAFLD, has increased in prevalence in recent years and has become the third major risk factor for the occurrence of hepatocellular carcinoma (HCC). Many studies have found that oxidative stress in NASH hepatocytes induces the production of a large number of inflammatory factors that induce gene expression and activate a self-targeted aggressive killing effect of CD8^+^ T-cells, which may be an important mechanism of NASH hepatocyte injury and progression to HCC. Whether Res and CuNPs can effectively inhibit oxidative stress, reduce inflammatory factor levels and reverse the inflammation associated with NAFLD is an important therapeutic question. The results suggest that aptamer-modified nanobubbles loaded with drugs and CuNPs have a significant anti-inflammatory effect.

The findings of this study are as follows: 1. NAFLD01 was verified to be a strong adapter that specifically binds to nonalcoholic fatty liver (NAFL) cells. 2. Aptamer-modified targeted nanobubbles were constructed to specifically bind to NAFL cells. 3. Synthesis of NAFLD-specific aptamer-modified targeted ultrasound nanobubbles loaded with Res and CuNPs was achieved. 4. The cytotoxicity and efficacy of aptamer-modified nanobubbles loaded with drugs and CuNPs in the treatment of NAFLD were demonstrated *in vitro*.

Until now, there has been no study on the combination of aptamer-modified nanobubble-loaded drugs and CuNPs for the treatment of NAFLD.

Currently, liver-targeted drug delivery systems (HTDDS) have been widely used against liver injury and liver diseases, such as liver fibrosis, and HCC. HTDDS not only prolong the blood circulation time of the drug, but also deliver the drug exclusively to the liver, reducing side effects. These systems use distinct ligands that can specifically recognize and interact with receptors that are particularly overexpressed on the surface of hepatocytes. Studies on more effective liver-targeting molecules is presently a research hotspot. However, because nucleic acid aptamers are more economical, stable, and have higher affinity and specificity than other ligands without immunogenicity, some researchers have found that NAFLD01 is an aptamer that specifically binds to NAFL cells. However, its targeting ability has not been generally recognized. Our research results affirm that aptamer-mediated nanobubbles can effectively target and bind to NAFL cells.

Our synthesized novel nanobubbles can address the inefficiency and side effects of Res treatment of NAFLD while simultaneously delivering CuNPs for a synergistic effect. Moreover, our findings suggest that Res and CuNPs effectively and synergistically inhibit NAFLD inflammation, and Apt-NBs@Res@Cu_5.4_O NPs are feasible as an effective therapeutic to reverse NAFLD at an early stage. Liver-targeted NAFLD-specific aptamer-mediated targeted ultrasound nanobubbles may become a safe and promising platform for the treatment of NAFLD and other liver diseases.

## 4 Conclusion

In this study, we successfully prepared NAFLD01 aptamer-functionalized ultrasound nanobubbles loaded with Res and CuNPs, named Apt-NBs@Res@Cu_5.4_O NPs, which can be used for the targeted treatment of NAFLD. A nanobubble concentration less than 310 nM can effectively treat early NAFLD, which provides a new approach for the synergistic treatment of NAFLD with aptamer-functionalized nanobubbles and nanoparticles.

## Data Availability

The original contributions presented in the study are included in the article/supplementary material, further inquiries can be directed to the corresponding author.
